# Supplementary material for: Enhancing inference of differential gene expression in metatranscriptomes from human microbial communities
Source: Nat Commun. 2026 Apr 21;17:5490. doi: 10.1038/s41467-026-71871-5 (PMC13284273; doi:10.1038/s41467-026-71871-5)
Supplement: Supplementary file 1 — Supplementary Information [file 41467_2026_71871_MOESM1_ESM.pdf]

SUPPLEMETARY NOTES

Supplementary Note 1: Validation of mock community properties and methodology

DNA and RNA from these mock communities were extracted, sequenced, and mapped to the constituent genomes to produce gene-level counts for *P. copri* and *E. coli* (**Supplementary Table 3;** see *Methods*). The fraction of DNA reads mapping to *P. copri* genes was balanced between the arabinan and glucose growth conditions and approximated the emulated relative abundances (**Supplementary Fig. 3a**). The same was not true for the fraction of RNA reads; *P. copri* genes constituted a higher fraction of RNA reads in mixtures from glucose cultures compared to arabinan cultures (**Supplementary Fig. 3b**). This increase in *P. copri* counts was distributed genome-wide and not driven by a few genes; this suggests a globally increased transcription rate for *P. copri* in glucose, though we note that other explanations are also possible (e.g., decreased mRNA degradation, nucleotide extraction biases). Additionally, this increase was not detectable in 100% *P. copri* mixtures because sequencing counts are relative and not absolute abundances[52]. DESeq2 inferred far more differentially expressed genes upregulated in glucose in the 50% *P. copri* samples compared to the 100% *P. copri* samples (**Supplementary Fig. 3c**).

To characterize method performance, we initially opted to control for these global transcriptional changes by rarefying MTX counts from the *P. copri* in glucose samples to balance the fraction of *P. copri* reads between conditions (**Supplementary Fig. 3d**, see *Methods*). We elected to do this because recent analyses have demonstrated that rarefaction robustly controls for confounding differences in sequencing effort[71-73]. Furthermore, diversity analyses are much more sensitive to loss of low abundance features than differential expression (i.e., loss of features detected at low abundance can substantially reduce the estimated diversity of the whole sample). In differential expression analyses, highly expressed genes will retain sufficient quantification to infer differential expression whereas lowly expressed genes at the limit of detection likely would not have met criteria for statistical significance.

Similarly, we assumed most users aim to determine differential expression analogous to what is obtained from a single-organism sample, where inference of genes upregulated in each condition is not influenced by global transcription rate changes (**Fig. 1b,c**). Without rarefaction, the definition of 'ground truth' upregulated genes would otherwise change between the pure (100%) *P. copri* and mixed samples, which is both undesirable for benchmarking purposes and would lead to false positives when comparing against the reference single-organism comparison. While this confounding feature was removed from the datasets for initial benchmarking, transcription rate changes at the organism-level are a possibility in real-world datasets and may signify bacterial growth and death[39] (see '*Benchmarking with confounding transcription rate changes*' below).

To validate the rarefaction approach, we verified that total sequencing effort was balanced across samples and that differential expression was identified evenly in both carbohydrate conditions for rarefied but not original counts (**Supplementary Fig. 3e-g**). We then verified that rarefaction did not bias differential expression results by selectively depleting counts from individual genes. After correcting for changes in the total number of reads per organism, both *P. copri* and *E. coli* gene-level RNA counts were well correlated in a near 1:1 ratio between original and rarefied counts (**Supplementary Fig. 3h**). This generalized across samples where rarefaction was applied, indicating that gene-level quantifications relative to each organism's total transcriptome were preserved (**Supplementary Fig. 3i,j**).

To verify that upstream processing of sequencing reads did not affect gene-level quantification, we also compared counts produced by an alignment-free tool, kallisto[74], against an alignment-based tool, bowtie2[75]. After correcting for differences in total mapped counts due to bowtie2's lower mapping rate, gene-level counts were well correlated, with a mean Pearson's R of 0.993 and a mean linear regression slope of 1.03. (**Supplementary Fig. 3k-m**). Based on these analyses, we opted to use rarefied kallisto counts given more even fractions of *P. copri* transcripts between conditions and the increased number of pseudoaligned reads compared to bowtie2 counts. To determine suitable probability distribution models for DNA and RNA counts, we fit various distributions to the observed counts data (see *Methods*). The results revealed that non-zero DNA counts for all genes were well-approximated by the gamma distribution (**Supplementary Fig. 3n**). RNA counts were approximated by the negative binomial distribution, although probabilities for low but non-zero expressed genes were underestimated. (**Supplementary Fig. 3o**).

Supplementary Note 2: Benchmarking with confounding transcription rate changes

We previously observed that *P. copri* genes constituted a higher fraction of RNA reads in mixtures from glucose cultures compared to those from arabinan cultures, resulting in biased inference of upregulation of genes in glucose (**Supplementary Fig. 3b,c**). Although this prevents a consistent definition of 'ground truth' gene upregulation between monocultures and mixtures, we leveraged this feature of the original sequencing counts from mock community datasets to determine whether statistical methods could correctly recover upregulated genes in both conditions despite confounding global transcription rate changes and produce results consistent with those obtained from sequencing a single-species sample. Given the larger fraction of RNA reads from *P. copri* in glucose, we additionally hypothesized that the

compositional nature of sequencing data (i.e., the non-independence of features measured as relative quantities[80-81]) would lead to lower counts per gene for *E. coli*, resulting in false positives despite the lack of differences in *E. coli* growth conditions.

We repeated our previous benchmarking scheme and again used DESeq2 to define true positive and true negative gene sets in the 100% *P. copri* mixtures (**Supplementary Fig. 6a; Supplementary Table 6a,b**). We similarly defined 2,627 true negative genes from the 100% *E. coli* samples (**Supplementary Fig. 6b; Supplementary Table 6c**). The *P. copri* true positive and true negative gene sets were used to calculate a *P. copri*-specific true positive rate and false positive rate (P-TPR, P-FPR), while the *E. coli* true negative gene set was used to calculate an *E. coli*-specific false positive rate (E-FPR). The *P. copri* metrics quantify consistency of results with sequencing of a single-organism sample while the *E. coli* FPR measures false positives due to compositional shrinkage of *E. coli* expression measurements due to increased transcription by *P. copri* in glucose. As before, sensitivity for differentially expressed *P. copri* genes decreased at low relative abundances, though taxon-scaled methods better maintained sensitivity in this case (**Supplementary Fig. 6c; Supplementary Table 7a**). Taxon-scaled methods better controlled the *P. copri* FPR, indicating that they were able to recover results that were unbiased by the background changes in transcription rate (**Supplementary Fig. 6c**). Compositional effects resulted in an inflated *E. coli* FPR for all methods without taxon-scaling, though this was less apparent when *E. coli* comprised 99% or more of the sample and where compositional effects from *P. copri* transcription were modest due to its low abundance (**Supplementary Fig. 6c**).

In comparisons where *P. copri* had different relative abundance in the two conditions, sensitivity again decreased in the presence of differential abundance (**Supplementary Fig. 6d; Supplementary Table 7b**). Taxon-scaling controlled both *P. copri* and *E. coli* false positive rates in all differential abundance comparisons as long as the organism was not absent (0%) in either group, further indicating the ability of taxon scaling to adequately control for changes in transcription rate and compositional effects. Again, MTXmodel failed to infer true positive differential expression when confounding differential abundance was present, though it also exhibited fewer false positives for both organisms (**Supplementary Fig 6d**). In comparisons where *P. copri* had low prevalence (i.e., was not present in all samples), DESeq2 demonstrated decreased P-TPR, P-FPR, and E-FPR, while MPRAnalyze and MTXmodel were unaffected by inclusion of uncolonized samples (**Supplementary Fig. 6e; Supplementary Table 7c**).

Supplementary Note 3: Validation of cross-feeding interactions *in vitro*

To validate this potential cross-feeding relationship identified in the mouse study, we advanced *P. copri* and *M. multacida* to *in vitro* mono- and coculture studies. Mono- and cocultures of *P. copri* and *M. multacida* were incubated in a defined medium[82] supplemented with 1% (w/v) arabinan, arabinose, or glucose as the sole carbohydrate (n=3 replicate cultures/condition; see *Methods*). When grown on arabinan, *P. copri* monocultures exhibited modest growth achieving an OD_{600} ~ 0.35 by 36 hours, while *M. multacida* alone did not demonstrate detectable growth. Coculture of the two exhibited increased total OD_{600} relative to each organism cultured independently (**Supplementary Fig. 8a; Supplementary Table 9**). In contrast, *P. copri* did not exhibit growth in glucose or arabinose in this time frame and cocultures of the two grew similarly to monocultures of *M. multacida*. We then used quantitative PCR (qPCR) to measure the abundance of each organism under each condition (n=3 biological replicates/condition; see *Methods*). Among substrates tested, only cocultures in arabinan supported simultaneous growth of both organisms and increased both *P. copri* and *M. multacida* cell density relative to either organism alone (**Supplementary Fig. 8b,c; Supplementary Table 10**). We advanced samples which exhibited detectable growth and yielded sufficient DNA and RNA to metagenomic and metatranscriptomic sequencing (**Supplementary Fig. 8d; Supplementary Table 11, 12**).

We then performed differential expression analysis to determine whether transcriptional changes by *M. multacida* in response to arabinan cross-feeding recapitulated the *in vivo* response to *P. copri* colonization. In simple comparisons of pure *Mitsuokella* samples grown in arabinose vs. glucose, MTXmodel and both the community-scaled and taxon-scaled DESeq2 approaches successfully inferred the expected upregulation of arabinose utilization genes in the pentose phosphate pathway (**Supplementary Fig. 8e; Supplementary Table 13**). The same differential expression of genes involved in glutamate, tryptophan, and glutamine biosynthesis observed *in vivo* were also detected by both DESeq2 approaches but not with MTXmodel. In comparisons of arabinan cocultures with glucose controls and along the time course of the experiment, MTXmodel provided high variance fold-change estimates and did not infer any significant genes in these pathways or the entire *M. multacida* genome (**Supplementary Fig. 8f,g**). DESeq2 detected expected upregulation of arabinose utilization genes in both comparisons and additionally inferred upregulation of tryptophan, glutamate, and glutamine biosynthesis at 72 h compared to 24 h (**Supplementary Fig. 8f,g**). Taxon-scaling resulted in relatively even differential expression inference for both conditions in the arabinan versus glucose comparison while community-scaling inferred a distribution of log-fold changes skewed towards upregulation in glucose, consistent with the differential abundance of *M. multacida* between the mono- and cocultures (violin plots, **Supplementary Fig. 8f**).

We applied targeted mass spectrometry to conditioned media samples to quantify tryptophan, glutamate, and glutamine and to determine whether transcriptional changes were associated with their availability. Compared to the *P. copri* monocultures in arabinan, the cocultures produced significantly more glutamate and depleted significantly more glutamine and tryptophan at 72 hours, indicating that *M. multacida* upregulation of these biosynthesis pathways was associated with low availability of tryptophan and glutamine and increased availability of glutamate (**Supplementary Fig. 8h**). In contrast, amino acid levels were not significantly different between endpoint cocultures and *M. multacida* monocultures for either monosaccharide condition (**Supplementary Fig. 8h**). Based on these results, we concluded that taxon-scaled DESeq2 was able to nominate cross-feeding interactions and downstream amino acid metabolic responses from our gnotobiotic animal studies which could then be validated *in vitro*.

SUPPLEMENTARY FIGURES

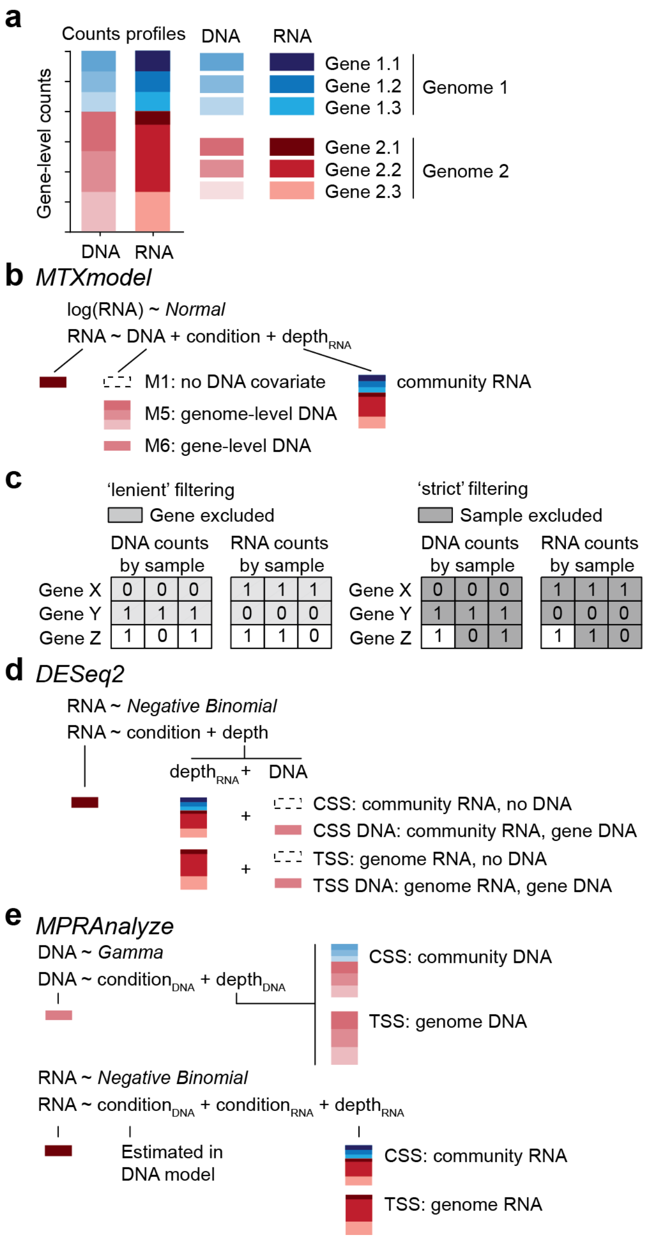

Supplementary Figure 1 – Metatranscriptomic differential expression statistical models. (a) Profiles of DNA and RNA counts for a sample containing two genomes (red and blue) with three genes each. **(b)** Statistical model used by MTXmodel under models M1, M5, and M6. **(c)** Zero-filtering approaches used by MTXmodel with 'lenient' gene-level filtering or 'strict' sample-level filtering. **(d)** Statistical model used by DESeq2 with either community- (CSS) or taxon-scaling (TSS) as well as with or without DNA covariates (DNA). **(e)** Statistical model used by MPRAnalyze for both DNA and RNA counts under community- and taxon-scaling. Abbreviations: M1, no DNA covariate; M5, genome-level DNA covariate; M6, gene-level DNA covariate; CSS, community-sum-scaling; TSS, taxon-specific-scaling. CSS DNA, community-scaling with DNA abundance normalization; TSS DNA, taxon-scaled with DNA abundance normalization.

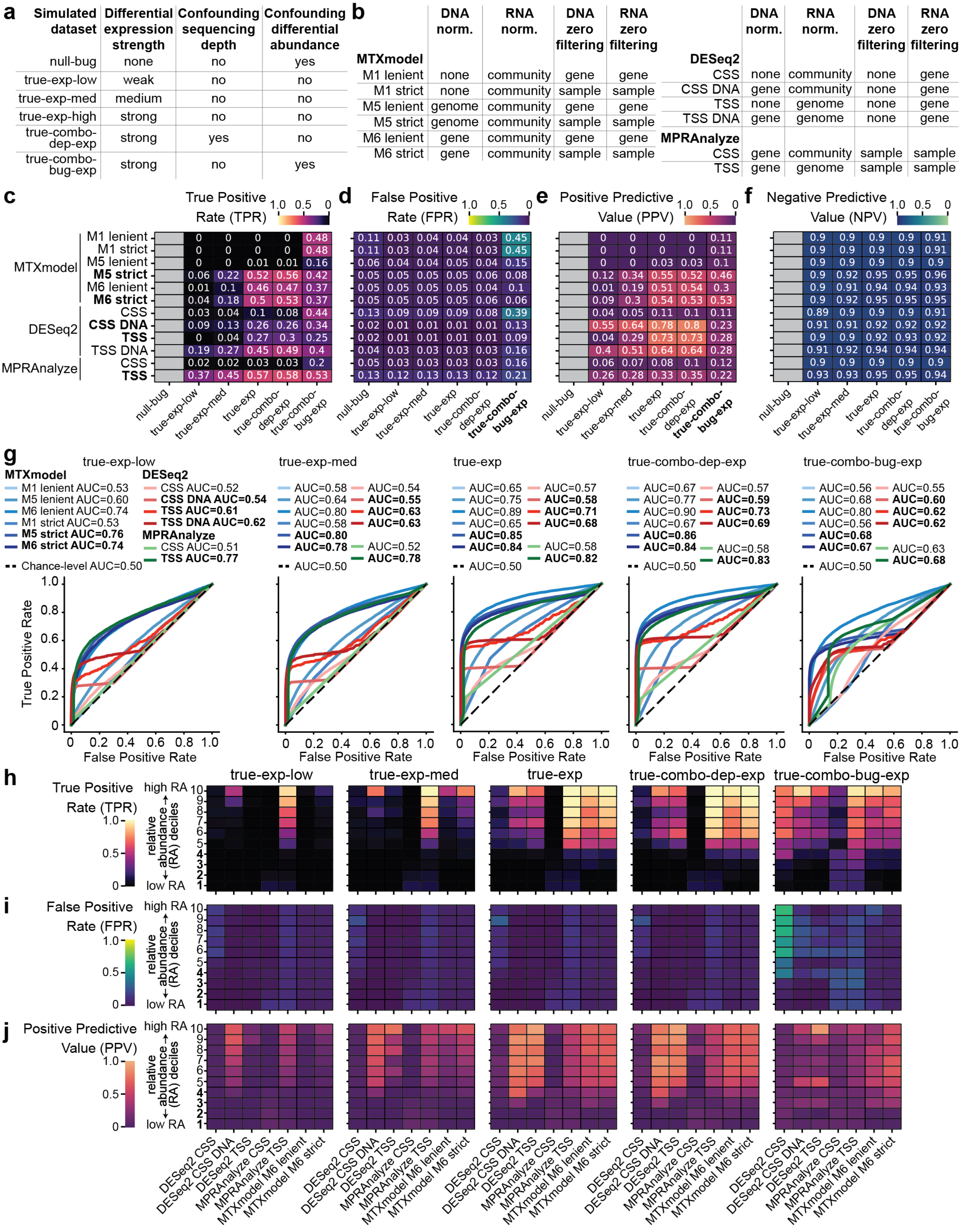

Supplementary Figure 2 – Statistical differential expression method benchmarking on simulated datasets for all methods tested. (a,b) Characteristics of simulated datasets (panel a) and differential expression methods and implementations tested (panel b). **(c-f)** True positive rate (TPR, or sensitivity; panel c), false positive rate (FPR, or 1-specificity; panel d), positive predictive value (PPV, or precision; panel e), and negative predictive value (NPV; panel f)

for MTXmodel, DESeq2, and MPRAnalyze across a range of parameter choices in six simulated datasets. Implementations and datasets specifically emphasized in the text are bolded. Metrics that are not defined for datasets lacking ground truth differential expression are grayed out. (**g**) Area under the curve (AUC) quantification and receiver operating characteristic curves for statistical methods in the five synthetic 'true-' datasets that included simulated differential expression. An AUC of 0.5 indicates the classification power of random guessing, with greater AUC indicating a greater ability to discriminate differentially expressed and non-differentially expressed genes. (**h-j**) True positive rate (panel h), false positive rate (panel i), and positive predictive value (panel j) for a subset of methods when applied to gene sets originating from organisms ranked and binned by their relative abundance (RA) in the simulated datasets. Abbreviations: M1, no DNA abundance covariates; M5, genome-level DNA covariates; M6, gene-level DNA covariates; CSS, community-sum-scaling; TSS, taxon-specific-scaling; DNA, DNA abundance normalization.

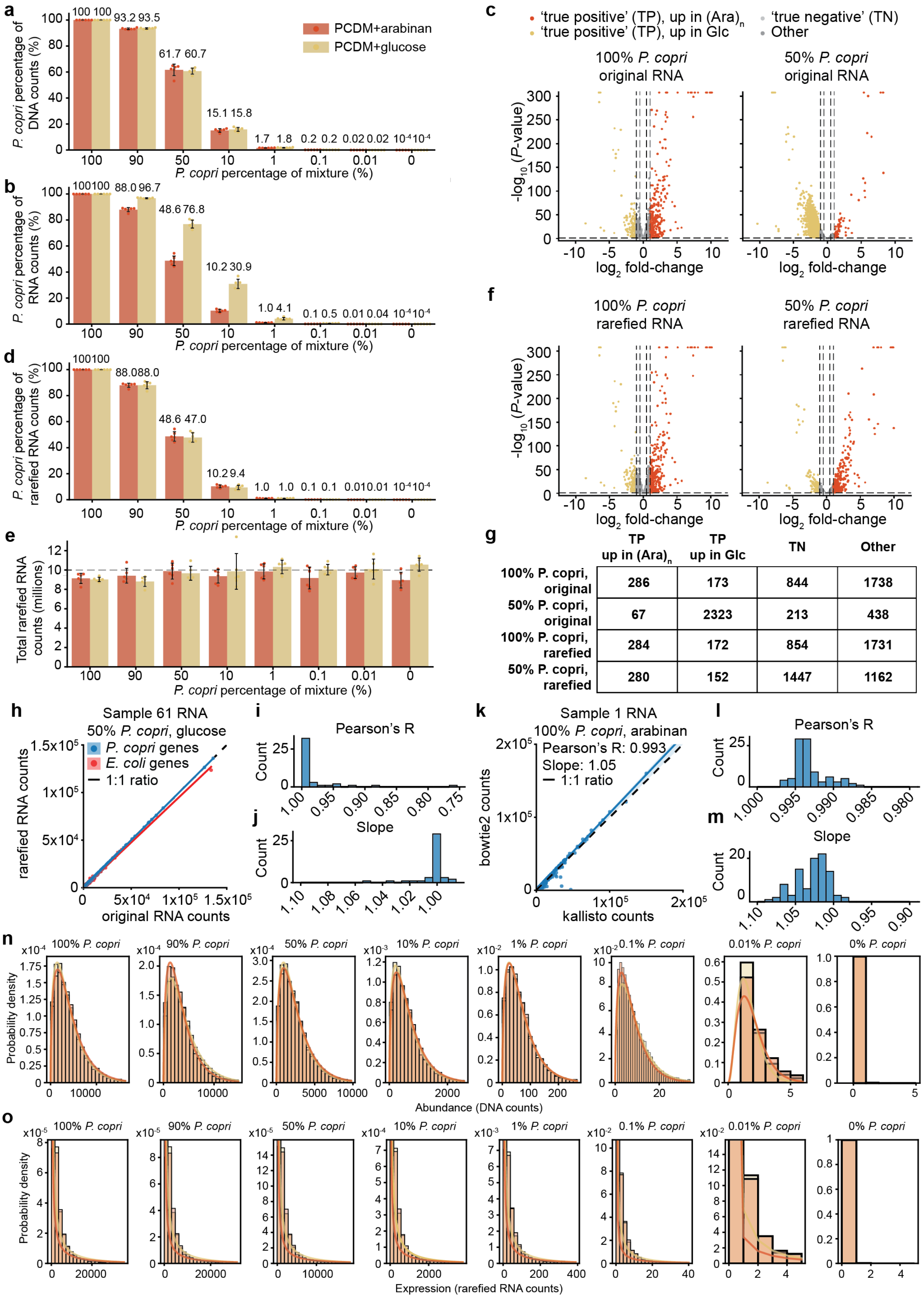

	TP up in $(Ara)_n$	TP up in Glc	TN	Other
100% P. copri, original	286	173	844	1738
50% P. copri, original	67	2323	213	438
100% P. copri, rarefied	284	172	854	1731
50% P. copri, rarefied	280	152	1447	1162

Supplementary Figure 3 – Validation and characterization of mock communities. (a,b) Percentage of non-rRNA microbial DNA (panel a) and RNA (panel b) reads mapping to *P. copri* for each mixture ratio and each condition. Mean ± standard deviation values are shown for the six replicates in each condition. **(c)** Volcano plots for results generated by community-scaled DESeq2 when applied to the 100% vs. 50% *P. copri* samples, demonstrating how increases in total transcription rate skew differential expression testing but are not readily detected in single-organism samples. (**d,e**) Percentage of non-rRNA microbial RNA reads mapping to *P. copri* (panel d) and total microbial non-rRNA reads (panel e) in mock community counts profiles after applying rarefaction to balance *P. copri* sequencing effort between conditions. (**f**) Volcano plots generated by community-scaled DESeq2 from rarefied counts profiles, indicating correction of varying skewness of differential expression results across abundance levels. (**g**) Number of genes meeting true positive (TP) and true negative (TN) log fold-change and adjusted *P*-value thresholds for 100% and 50% *P. copri* comparisons using the original and rarefied counts. Directionality and number of TP genes are maintained between datasets using rarefied but not original counts. (**h**) Representative regression plot of *P. copri* (blue) and *E. coli* (red) gene-level RNA counts after adjusting for total counts for each organism for one sample containing 50% of each organism. The dashed black line shows a 1:1 ratio, indicating preservation of gene-level relative representation within each organism's total transcriptome. (**i,j**) Histograms of Pearson's correlation coefficients (Pearson's R; panel i) and ordinary least squares linear regression slopes (panel j) comparing gene-level RNA counts after adjusting for organism-level sequencing effort (as in panel h) across the 48 glucose-conditioned samples where rarefaction was applied. **(k)** Representative regression plot of kallisto pseudoaligned counts vs. bowtie2 aligned counts for all *P. copri* genes in one sample after adjusting for total mapped reads to account for the lower mapping rate of bowtie2. Pearson's correlation coefficient and the slope of a simple linear model are annotated. **(l,m)** Histograms of Pearson's correlation coefficients (panel l) and linear model slopes (panel m) for correlations between kallisto and bowtie2 counts for all 96 samples. (**n,o**) Non-zero DNA counts (panel n) and all RNA counts (panel o) across genes for each of the six replicate samples for each mixture ratio and carbohydrate condition. The probability density function for gamma distributions fit by maximum likelihood estimation for each condition are shown as solid lines in panel n. The probability mass functions of negative binomial distributions fit by the method of moments are shown as solid lines in panel o. PCDM, *P. copri* defined medium, adapted from a previous publication[82].

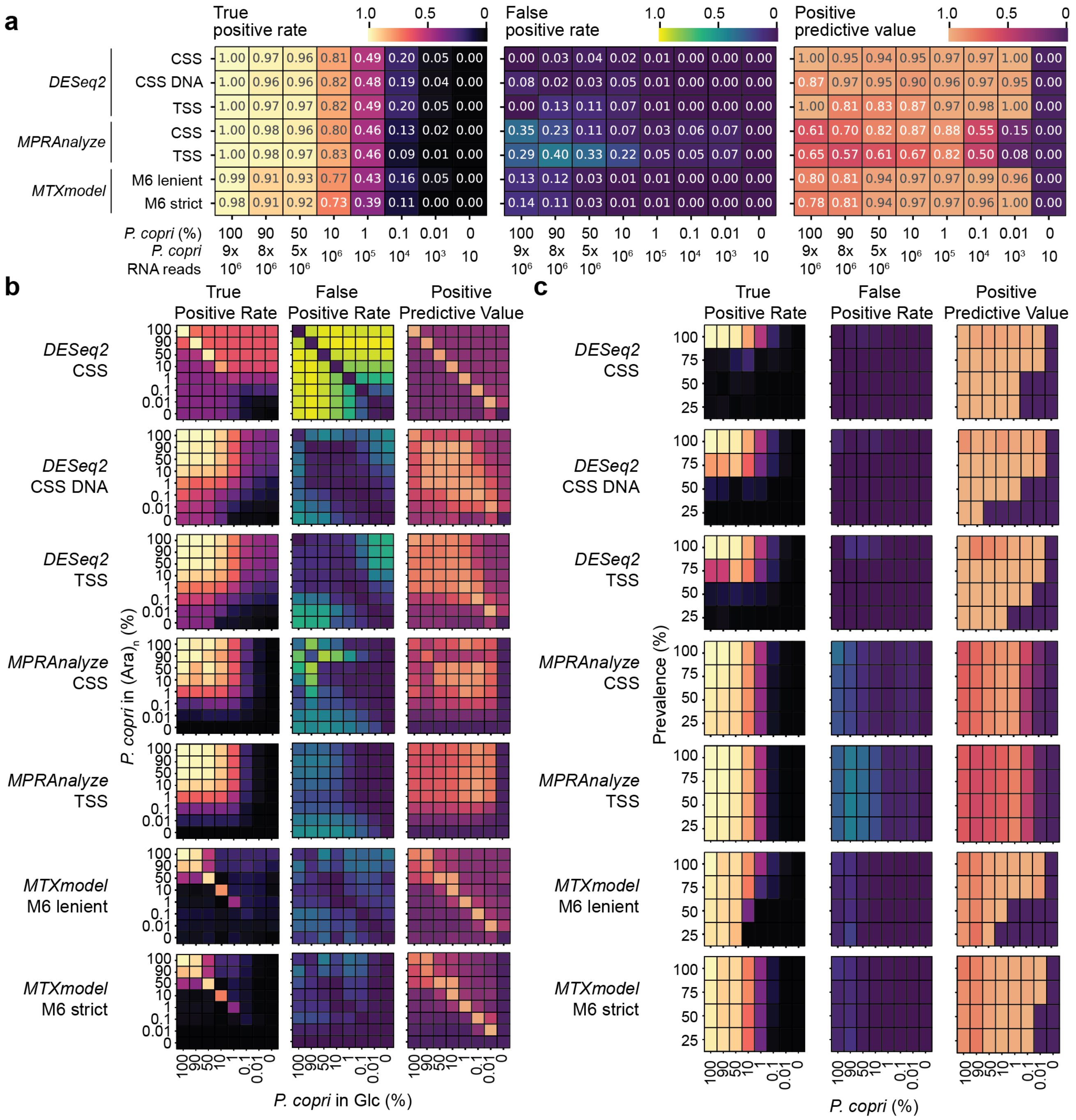

Supplementary Figure 4 – Sensitivity, specificity, and precision of datasets from mock communities. (**a**) True positive rate (TPR), false positive rate (FPR), and positive predictive value (PPV) for benchmarked methods when analyzing the different mixture ratios of *P. copri*, from highest to lowest relative abundance. (**b**) TPR, FPR, and PPV for methods in comparisons with varying strengths of differential abundance, emulated by performing all pairwise comparisons between mixture ratios. (**c**) TPR, FPR, and PPV of methods when emulating decreasing prevalence by including either 0, 2, 6, or 18 100% *E. coli* samples with the six *P. copri*-containing samples in each group. Abbreviations: $(Ara)_n$, arabinan; Glc, glucose; TP, true positive; TN, true negative; CSS: community-sum-scaling; CSS DNA: community-sum scaling with DNA abundance normalization; TSS, taxon-specific-scaling; TPR, true positive rate; FPR, false positive rate; PPV, positive predictive value.

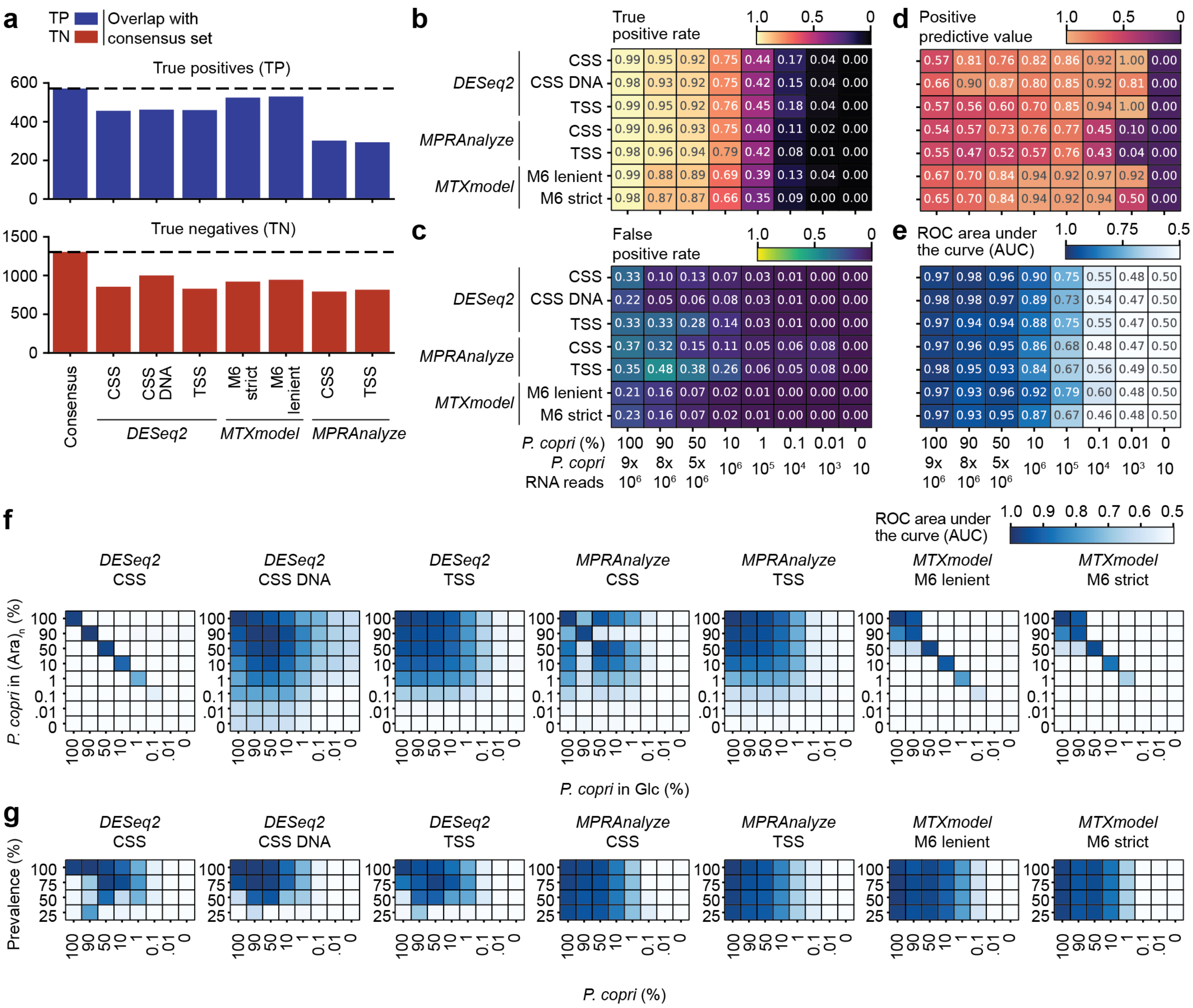

Supplementary Figure 5 – Benchmarking on mock communities using consensus sets of true positive and true negative genes. (a) Consensus true positive genes (n=571) and true negative genes (n=1,304) were defined as the union across the true positive and true negative gene sets determined using individual methods in **Fig. 3c.** Dashed lines indicate the size of the consensus sets and each method's intersection with the consensus sets are shown in colored bars. (**b-e**) True positive rate (panel b), false positive rate (panel c), positive predictive value (panel d), and ROC AUC (panel e) calculated using the consensus gene sets for benchmarked methods when analyzing the different mixture ratios of *P. copri*, from highest to lowest relative abundance. (**f,g**) ROC AUC quantification in comparisons confounded by differential abundance (panel f) and varying prevalence of *P. copri* (panel g). Abbreviations: TP, true positive; TN, true negative; CSS: community-sum-scaling; CSS DNA: community-sum scaling with DNA abundance normalization; TSS, taxon-specific-scaling; TPR, true positive rate; FPR, false positive rate; PPV, positive predictive value; ROC, receiver operating characteristic; AUC, area under the curve quantification.

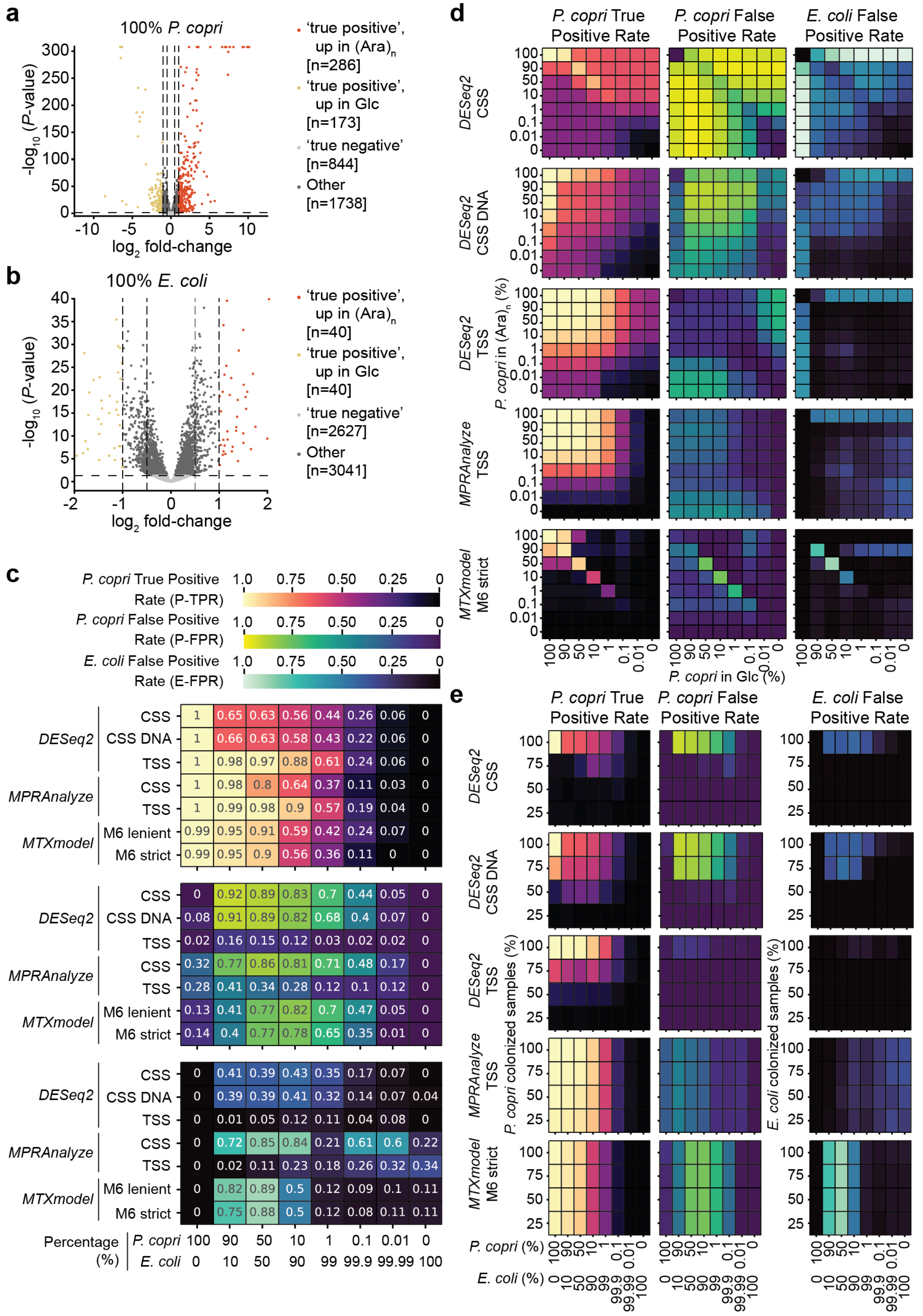

Supplementary Figure 6 – Benchmarking on datasets from mock communities with confounding transcription rate changes and compositional effects. (a,b) True positive and true negative gene sets defined by DESeq2 for 100% *P. copri* samples (panel a) and for 100% *E. coli* samples (panel b). (**c**) True positive rate for the *P. copri* TP gene set (P-TPR), false positive rate for the *P. copri* TN gene set (P-FPR), and false positive rate for the *E. coli* TN gene set (E-FPR) for each of the methods for each mixture ratio, in order of descending *P. copri* relative abundance. (**d**) P-TPR, P-FPR, and E-FPR for datasets with emulated differential abundance for DESeq2 with community- or taxon-scaling, MPRAnalyze with taxon-

scaling, and MTXmodel using gene-level DNA covariates. (**e**) P-TPR and P-FPR for datasets with emulated non-colonization by inclusion of 100% *E. coli* samples in each group. Abbreviations: CSS, community-sum-scaling; CSS DNA, community-sum scaling with DNA abundance normalization; TSS, taxon-specific-scaling; P-TPR, *P. copri* true positive rate; P-FPR, *P. copri* false positive rate; E-FPR, *E. coli* false positive rate.

a

Simulated dataset	Sample size	Complexity (n species)	Differential expression strength	Confounding sequencing depth	Confounding differential abundance
n12-true-exp-low	12	100	weak	no	no
n12-true-exp-med	12	100	medium	no	no
n12-true-exp-high	12	100	strong	no	no
n12-true-combo-dep-exp	12	100	strong	yes	no
n12-true-combo-bug-exp	12	100	strong	no	yes

b

Simulated dataset	Sample size	Complexity (n species)	Differential expression strength	Relative abundance (%)	
				BUG0001	BUG0002
sim-Pco100	12	2	strong	100	0
sim-Pco90	12	2	strong	88	12
sim-Pco50	12	2	strong	48	52
sim-Pco10	12	2	strong	9.8	90.2
sim-Pco1	12	2	strong	1.0	99
sim-Pco01	12	2	strong	0.10	99.9
sim-Pco001	12	2	strong	0.01	99.99
sim-Pco0	12	2	strong	0	0

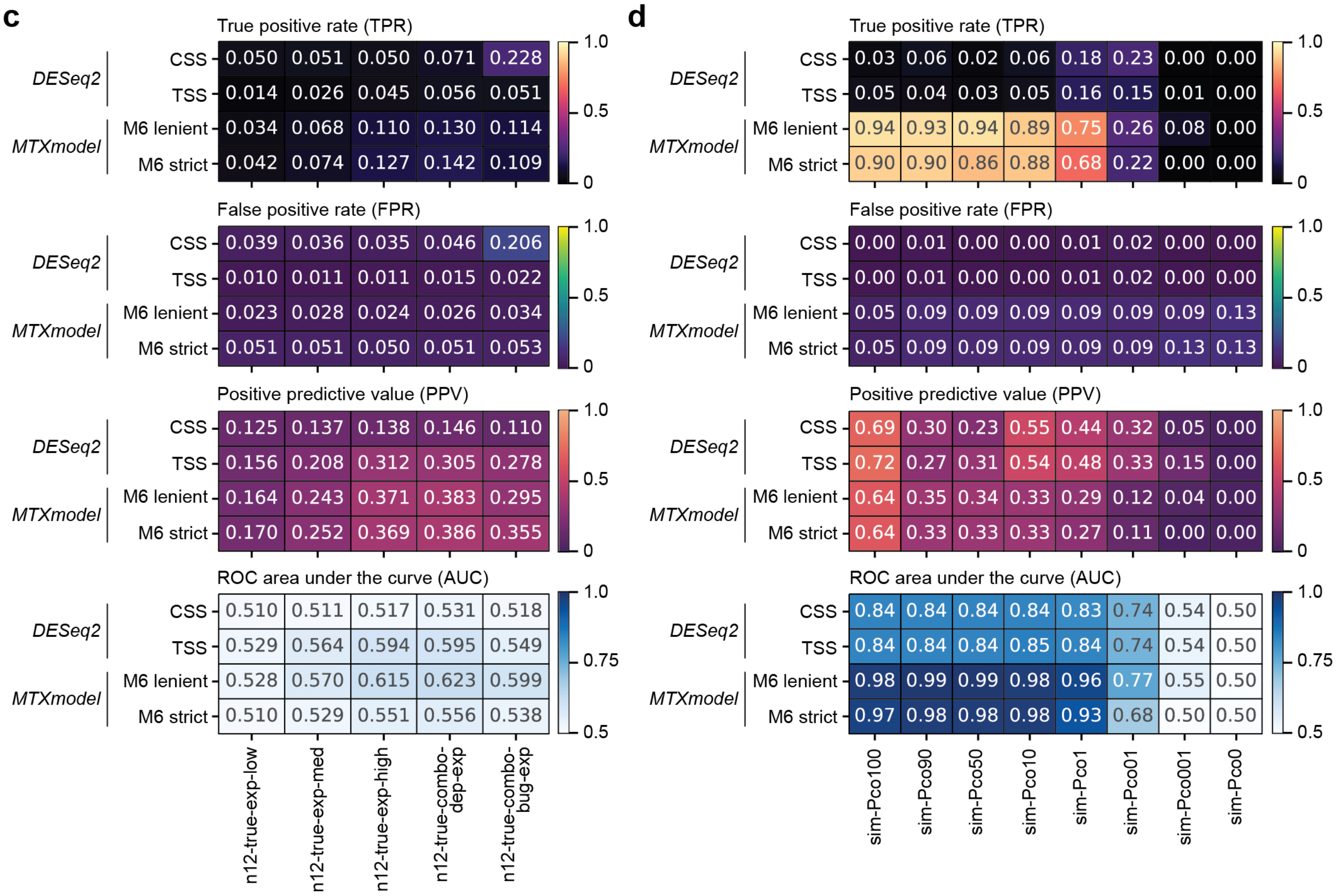

Supplementary Figure 7 – Differential expression method benchmarking on simulated datasets with analogous properties to mock communities. (a,b) Characteristics of simulated datasets with sample size identical to mock community datasets (n=6 simulated samples per condition with 100 species; panel a) and simulated datasets with both sample size, complexity, and relative abundances based on mock communities (n=6 simulated samples per condition with 2 species; panel b). (**c,d**) True positive rate, false positive rate, and positive predictive value calculated at a significance threshold of 0.05, as well as ROC AUC calculated across significance thresholds for the 100 species synthetic communities (panel c) and the two-member synthetic communities (panel d). Abbreviations: CSS: community-sum-scaling; TSS, taxon-specific-scaling; TPR, true positive rate; FPR, false positive rate; PPV, positive predictive value; ROC, receiver operating characteristic; AUC, area under the curve.

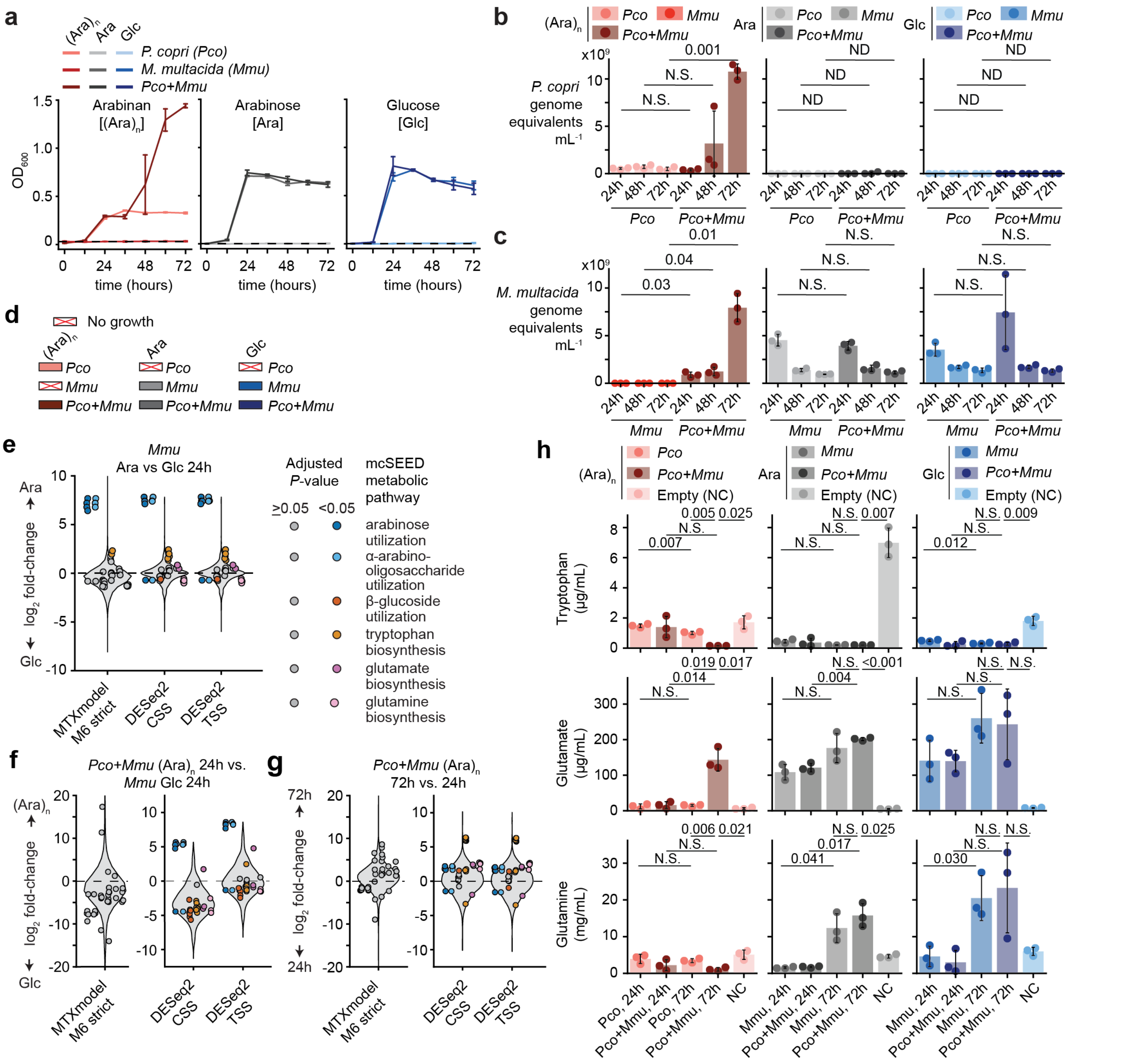

Supplementary Figure 8 – *In vitro* validation of arabinan cross-feeding between *P. copri* and *M. multacida*. (a) Growth of monocultures and cocultures of *P. copri* (*Pco*) and *M. multacida* (*Mmu*) in PCDM supplemented with either 1% arabinan, arabinose, or glucose (n=3 per inoculated organism(s) per growth condition per timepoint). **(b,c)** qPCR quantification of *P. copri* (panel b) and *M. multacida* (panel c) in mono- and cocultures. **(d)** Experimental design for samples advanced to sequencing and mass-spectrometry based on growth and qPCR quantification in panels a-c. **(e-g)** Differential expression results for *M. multacida* genes predicted to be involved in arabinose, alpha-arabinoside, and beta-glucoside utilization, as well as biosynthesis of glutamate, glutamine, and tryptophan as reported by the three methods using an adjusted *P*-value threshold of 0.05. Violin plots represent the distribution of log2 fold-change across all genes in the *M. multacida* genome; points represent differential expression test results for individual genes from select metabolic pathways, horizontally grouped by pathway and colored if they were significantly differentially expressed with a given method. The dashed line indicates a fold-change of 1 (no change in expression). Comparisons between arabinose and glucose *M. multacida* monocultures are shown in panel e, comparisons between arabinan cocultures and *M. multacida* in glucose are shown in panel f, and comparisons between 72 h and 24 h arabinan cocultures are shown in panel g. **(h)** Mass-spectrometric quantification of glutamate, glutamine, and tryptophan from conditioned media collected from mono- and cocultures every 24h for each condition. Mean ± standard deviation values are shown in panels a-c and h. *P*-values were

calculated by a two-sided Welch T-test of independent samples with unequal variance in panels b, c, and i, and the specified differential expression methods in panels e-g. Abbreviations: (Ara)n, arabinan; Ara, arabinose; Glc, glucose; *Pco*, *P. copri*; *Mmu*, *M. multacida*; N.S., not statistically significant (Benjamini-Hochberg adjusted P-value > 0.1); ND no detection in both groups (quantification lower than inoculum input); NC, no inoculum negative control.

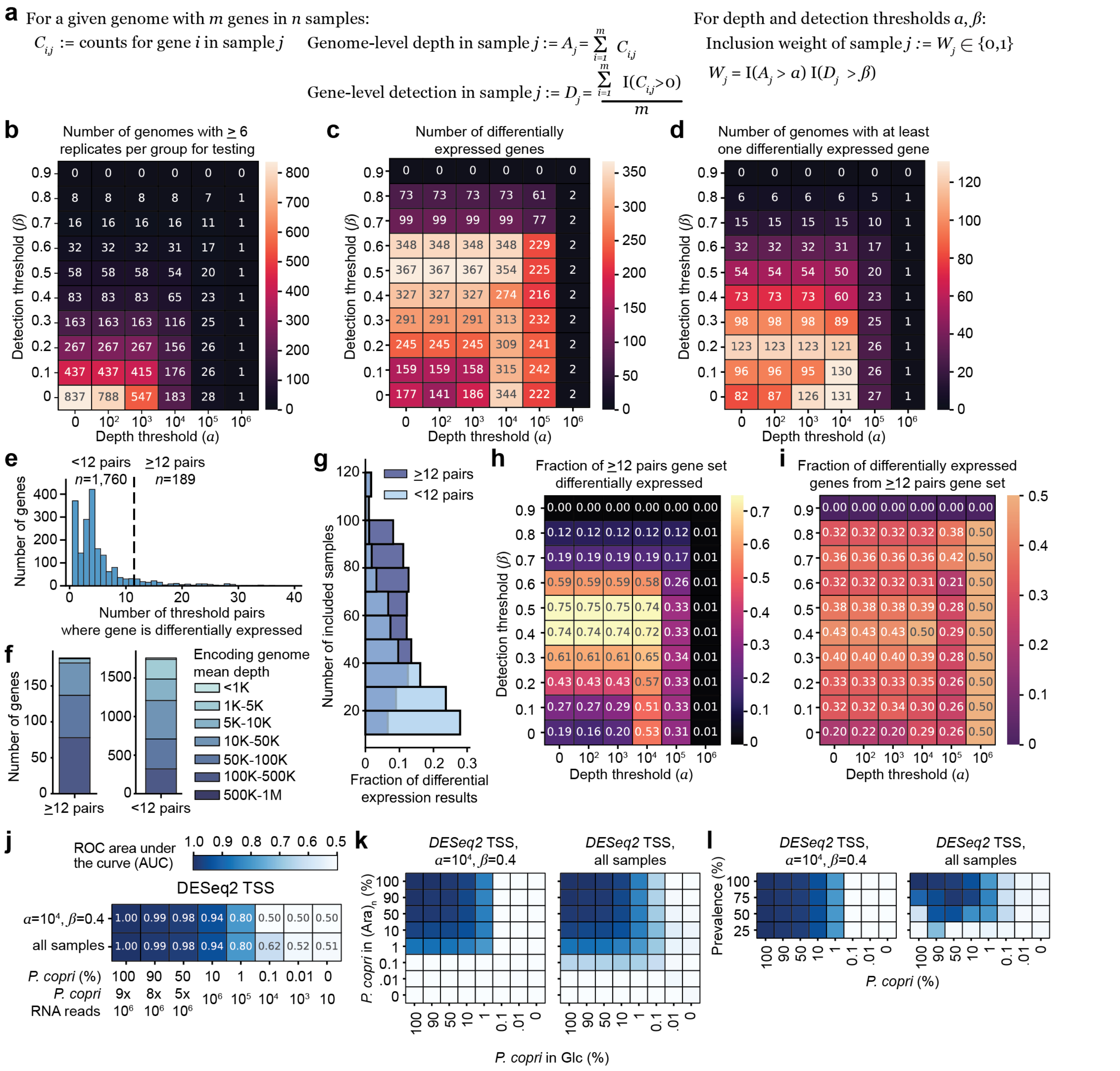

Supplementary Figure 9 – Parameter sweep of genome-level depth and gene-level detection thresholds for sample inclusion in differential expression analysis of the human study. (a) Definition of genome-level depth and gene-level detection metrics for a given sample, as well as inclusion of a sample at depth and detection thresholds α and β. Samples are filtered separately for each genome's differential expression analysis if their genome-level depth is not greater than α or if their gene-level detection is not greater β. **(b-d)** The number of genomes with sufficient samples for testing for differential expression (panel b), the number of differentially expressed genes (panel c), and the number of genomes with at least one differentially expressed gene (panel d) across a range of depth (α) and detection thresholds (β). **(e)** The distribution of the number of threshold pairs where a gene was significantly differentially expressed for all genes which were significant in at least one set of thresholds (1,949 genes). A set of 189 genes was defined as having inferred differential expression in at least 12 (20%) of the threshold pairs. **(f,g)** The average depth of genomes encoding genes (panel f) and the number of samples included for differential expression analysis (panel g) for genes recovered in at least 12 threshold pairs or fewer than 12 threshold pairs. **(h,i)** The fraction of this set of genes which was inferred as differentially expressed (panel h) and the fraction of all significant differential expression results from this set of genes (panel i) across the range of depth (α) and detection thresholds (β). Statistical significance was defined using a cutoff of 0.25 for P-values adjusted across all genes from all genomes with sufficient replicates for analysis (up to 1,929,056 genes

with no sample filtering). **(j-l)** ROC AUC for differentially expressed genes in mock communities in comparisons with no differential abundance (panel j), differential abundance (panel k), or low prevalence (panel l) for taxon-scaled DESeq2 either including all samples or filtering samples using depth and detection thresholds of $\alpha=10^4$ *and* $\beta=0.4$. Abbreviations: ROC, receiver operating characteristic; AUC, area under the curve; TSS, taxon-specific-scaling.
