# Supplementary material for: Enhancing inference of differential gene expression in metatranscriptomes from human microbial communities
Source: Nat Commun. 2026 Apr 21;17:5490. doi: 10.1038/s41467-026-71871-5 (PMC13284273; doi:10.1038/s41467-026-71871-5)
Supplement: Supplementary file 2 — Description of Additional Supplementary Files [file 41467_2026_71871_MOESM2_ESM.docx]

**SUPPLEMENTARY DATA FILES**

**File Name**: Supplementary Data 1

**Description**: Supplementary Datasets 1-16. Source and supporting data for figures.

**Supplementary Dataset 1**. **Differential expression method performance on six simulated datasets. (a)** Simulated dataset characteristics. **(b)** Performance metrics by method, implementation, and simulated dataset. **(c)** Characteristics of relative abundance deciles for five datasets with simulated differential expression. **(d)** Performance metrics for relative abundance deciles by method and implementation for each simulated dataset.

**Supplementary Dataset 2. Sequencing metadata for mock communities. a)** Bacterial strains used in mock communities. **(b)** Sequencing metadata - metagenomic (DNA) libraries. **(c)** Sequencing metadata - metatranscriptomic (RNA) libraries.

**Supplementary Dataset 3. Mapping statistics for mock community datasets. (a)** Mapping statistics - metagenomic (DNA) libraries with and without rarefaction. **(b)** Mapping statistics - metatranscriptomic (RNA) libraries with and without rarefaction. **(c)** Mapping statistics - metagenomic (DNA) libraries quantified by kallisto and bowtie2. **(d)** Mapping statistics - metatranscriptomic (RNA) libraries quantified by kallisto and bowtie2.

**Supplementary Dataset 4. Differentially expressed gene sets used for benchmarking in Fig. 3. (a)** True positive genes. (**b)** True negative genes.

**Supplementary Dataset 5. Performance metrics for benchmarking on mock communities described in Fig. 3. (a)** Performance metrics by method for comparisons with no differential abundance. **(b)** Performance metrics by method for comparisons with emulated differential abundance. **(c)** Performance metrics by method for comparisons with emulated varying prevalence.

**Supplementary Dataset 6. Differentially expressed gene sets used for benchmarking in Supplementary Fig. 6. (a)** *P. copri* true positive genes. **(b)** *P. copri* true negative genes. **(c)** *E. coli* true negative genes.

**Supplementary Dataset 7. Performance metrics for benchmarking on mock communities described in Supplementary Fig. 6. (a)** Performance metrics by method for comparisons with no differential abundance. **(b)** Performance metrics by method for comparisons with emulated differential abundance. **(c)** Performance metrics by method for comparisons with emulated varying prevalence.

**Supplementary Dataset 8. Differential expression analysis of gnotobiotic mouse datasets in Fig. 4. (a)** Organism-level differential expression summary. **(b)** Differential gene expression in *Mitsuokella multacida.*

**Supplementary Dataset 9. OD_600_ data for mono- and cocultures described in Supplementary Fig. 8. (a)** OD_600_ measurements for all samples and controls.

**Supplementary Dataset 10. qPCR data for mono- and cocultures described in Supplementary Fig. 8. (a)** qPCR assay for *P. copri.* **(b)** qPCR assay for *M. multacida.*

**Supplementary Dataset 11. Sequencing metadata for in vitro cross-feeding experiments. (a)** Bacterial strains used in cross-feeding experiments. **(b)** Sequencing metadata - metagenomic (DNA) libraries. **(c)** Sequencing metadata - metatranscriptomic (RNA) libraries.

**Supplementary Dataset 12. Mapping statistics for *in vitro* cross-feeding experiments. (a)** Mapping statistics - metagenomic (DNA) libraries. **(b)** Mapping statistics - metatranscriptomic (RNA) libraries.

**Supplementary Dataset 13. *M. multacida* differentially expressed genes in cross-feeding experiments. (a)** 24h arabinose monoculture vs 24h glucose monoculture. **(b)** 24h arabinan coculture vs 24h glucose monoculture. **(c)** 72h arabinan coculture vs 24h arabinan coculture.

**Supplementary Dataset 14. Parameter sweep of depth and detection thresholds for analysis of the human study. a)** Differentially expression testing metadata across depth and detection thresholds.

**Supplementary Dataset 15. Definition of differentially expressed gene sets by their recovery across the parameter sweep. (a)** Genes recovered in at least 12 (20%) of the depth and detection threshold pairs. **(b)** Genes recovered in fewer than 12 (20%) of the depth and detection threshold pairs. (**c)** Recovery of the >12 threshold pairs gene set across depth and detection thresholds.

**Supplementary Dataset 16. Differentially expressed genes with or without depth-detection filtering. (a)** Differentially expressed genes using *α*=10^4^, *ß*=0.4. **(b)** Differentially expressed genes without sample filtering. **(c)** MTXmodel differentially expressed genes.

**File Name**: Supplementary Data 2

**Description**: Supplementary Datasets 17-24. Supplementary Datasets 17-24. Processed counts datasets, metadata, benchmarking gene sets, and metabolic gene annotations for mock communities.

**Supplementary Dataset 17**. Processed DNA counts for all 96 mock community samples.

**Supplementary Dataset 18**. Processed RNA counts for all 96 mock community samples, with rarefaction applied to control total transcriptional output differences between conditions.

**Supplementary Dataset 19**. Processed RNA counts for all 96 mock community samples, original counts data without rarefaction.

**Supplementary Dataset 20**. Sample metadata.

**Supplementary Dataset 21**. *P. copri* true positive genes (rarefied counts, for use with Supplementary Datasets 17 and 18).

**Supplementary Dataset 22**. *P. copri* true negative genes (rarefied counts, for use with Supplementary Datasets 17 and 18).

**Supplementary Dataset 23**. mcSEED genome annotations for *P. copri.*

**Supplementary Dataset 24**. mcSEED genome annotations for *E. coli.*
