# Supplementary material for: Enhancing inference of differential gene expression in metatranscriptomes from human microbial communities
Source: Nat Commun. 2026 Apr 21;17:5490. doi: 10.1038/s41467-026-71871-5 (PMC13284273; doi:10.1038/s41467-026-71871-5)
Supplement: Supplementary file 6 — Transparent Peer Review file [file 41467_2026_71871_MOESM6_ESM.pdf]

nature portfolio

# Peer Review File

# Enhancing inference of differential gene expression in metatranscriptomes from human microbial communities

Corresponding Author: Dr Jeffrey Gordon

**This file contains all reviewer reports in order by version, followed by all author rebuttals in order by version.**

Version 0:

Reviewer comments:

Reviewer #1

(Remarks to the Author)
Lee and colleagues present a tour de force evaluation of computational methods for microbial community differential expression (DE) from metatranscriptomics (MTX) with a strong focus on in vitro and gnotobiotic validation. The authors first expand recent evaluations based solely on synthetic data to a wider array of statistical methods, including several new variants of the DEseq2 method that is ubiquitous in single-organism DE analysis. Then, in the main new contribution of the paper, they repeat this evaluation using a range of mock communities constructed to replicate real-world challenges to MTX DE (e.g. confounding with species abundance fluctuations) and a gold standard based on monoculture DE experiments. In follow-up analyses, they then apply lessons from these evaluations to a real-world gnotobiotic experiment and show how the methods can be potentially adapted for better performance in human microbiome epidemiology.

Overall, I think this is an excellent piece of work. Interest in and methods for MTX DE have been exploding in recent years, but there are so many things that go wrong in its execution and interpretation, that more careful benchmarking work like this manuscript is greatly needed. I strongly appreciated the authors' focus on benchmarking using mock community data vs. purely synthetic data, which requires a lot of effort and fills in gaps from a purely synthetic approach. The proposed methodological additions to DEseq2 were also very interesting and I appreciated the authors' summary recommendations. The manuscript is well-written but long, although it's also hard to imagine removing any of the major sections without harming the story. My major comments are focused on potential under-discussed weaknesses of the authors' mono- vs. community-DE assumptions and some unexpected aspects of the between-methods comparisons.

MAJOR COMMENTS

* I will say from the outset that I thought the authors' approach of using monoculture DE to establish a gold standard for community DE was very clever, and I can't really imagine a better way of doing this. That said, unless I'm missing something, there's a huge assumption here that the monoculture bug doesn't change its transcriptional program in the community context (i.e. that DE'ed vs. non-DE'ed genes persist as a reasonable gold standard from monoculture to community context). Indeed, some of the findings of the gnotobiotic experiment underscore that bugs do different things when community structure changes. I think this can be largely addressed as part of an expanded "strengths and weaknesses" discussion vs. purely synthetic approaches (which are more controlled but less realistic). If there IS any way to estimate the stability of the DE lists, that would be interesting. Many other aspects of potential monoculture vs. community changes (e.g. effect of total transcript pool) WERE very carefully controlled in the study - well done.

* I was surprised by some of the differences in method performance between the synthetic and mock community data (especially variability in robustness to species abundance effects), and I wonder to what extent this was influenced by the difference in synthetic vs. mock sample sizes? The synthetic datasets had N=100 samples whereas it sounded like the mock communities had N=6 replicates, which I assume means N=6 vs. 6 in testing? (Notably these details were hard to find in the text, despite their importance.) I don't expect the authors to scale up to N=100 mock community replicates, but they could easily subsample the synthetic data down to N=12 (or whatever is appropriate) for a fairer comparison. Incorporating more AUC-based comparisons might also help, as this softens the influence of methods' dependence on N for achieving $p<0.05$.

* I'm confused by the behavior of DESeq2 CSS DNA in the Fig. 1f ROC plots. It seems to be near optimal in 4/5 plots, but that's not reflected in the nearby heatmaps (where it's less of an outlier). There was a note in methods about DESeq2 AUCs

being inflated that seemed like it might be related (in addition to being generally concerning from a benchmarking standpoint).

MODERATE COMMENTS

* Related to the major comment above about monoculture vs. community DE, I appreciated that the authors didn't use a single method to establish monoculture DE, as this would have some of the same simulation method vs. analysis method concerns that the authors criticized in the context of purely synthetic evaluation. That said, it is worth discussing how focusing on a consensus monoculture DE genes set might affect community-level evaluations, and indeed if this consensus might miss certain classes of true biological DE.

* When reporting benchmarks / making recommendations based on species abundance, I strongly recommend reporting an estimated coverage depth instead of (or alongside) relative abundance (RA). The former does not depend on sequencing depth, while the latter does, and sequencing depth was not always immediately referenced alongside RA values for context (and would require additional math from the reader to translate anyhow). As context, I strongly suspect that something like loss of sensitivity at low relative abundance has more to do with (e.g.) only 50% of the genome being covered, and if you increased depth by 10x (without changing relative abundance) you might see the problem alleviated. If that's NOT the case, and (say) RA effects have more to do with community complexity vs. genome coverage, THAT is worth emphasizing.

* The fact that the DEseq2-inspired methods only seem to be optimal at very high prevalence is limiting from a human epidemiology POV. I appreciated that the authors noted this at least once (i.e. stressing that these methods are best for in vitro / gnotobiotic work), but that wasn't super consistent with those methods coming across as the authors' main recommendation for MTX DE (assuming I was reading into that correctly?). The human microbiome application at the end gets into this some with the sample:genome filtering suggestions, which were interesting and made sense, but by that point the authors were operating without a synthetic OR mock gold standard for unbiased evaluation.

* If a difference in Ns does not explain the synthetic vs. mock benchmarking differences (see major comment above), it would be nice to at least speculate on what MIGHT explain the differences for the purposes of improving the construction of future synthetic datasets and/or MTX DE methods.

* I applaud the authors for the amount of benchmarking detail in Fig. 2 and 3 (and their SI analogs). However, it might be nice to also find a couple of views that would facilitate easier "at-a-glance" comparison between the various methods. For example, maybe a couple of representative AUC bar plots in Fig. 3 that illustrate the authors' core messages about robustness to differential abundance vs. prevalence.

MINOR COMMENTS

* It would be nice to include a small description of the meaning of the different synthetic datasets to help with interpretation (in the spirit of the tables in Fig. 1a).

* FYI Submitting the manuscript single-spaced was not ideal for review. Thankfully the original DOCX file was also provided and could be adjusted for a better experience.

(Remarks on code availability)
Methods description and data/code reporting all seemed solid, but I did not have the bandwidth to review the repository contents in detail.

Reviewer #2

(Remarks to the Author)
This manuscript compares metatranscriptome analysis methods using simulated data and mock communities. The authors conclude that taxon-scaled DESeq2 is the preferred method for simple microbial communities based on the mock community study. This finding is effectively supported by validation using intestinal metatranscriptomes from gnotobiotic mice. The study is comprehensive, well-designed, and its conclusions are convincing. This benchmarking work is valuable to the research community. I support its publication in Nature Communications.

There are some issues I want the authors to address:

1. At the core of this manuscript is a benchmarking study using in vitro mock communities. The authors conclude that taxon-scaled DESeq2 performs best among the tested methods. However, the gold standard for comparison was also established using DESeq2 on a single-species "community" of P. copri—which, by its nature, is inherently "taxon-scaled." This raises a concern about potential methodological bias, as the gene set used for evaluation was selected by the very method that is regarded as the top performer. I recommend the authors dedicate a section of the discussion to explicitly address this concern in their manuscript.

2. While the mock community experiments involve mixtures of P. copri and E. coli at different ratios, the simulation studies do not appear to include a directly analogous dataset. Specifically, a simulated community replicating the simple two-species composition and known ratios used in the mock experiments would provide the most straightforward comparison. I recommend that the authors address this point in the manuscript, explaining their rationale for not including this most directly

comparable simulation scenario in their study.

3. The authors employ rarefaction to address global RNA expression changes. This method should be introduced immediately following the discussion of genomic abundance (lines 78–87), as the two issues are presented as equally important in Figure 1b.

4. The author's presentation blurs the boundaries between sections, making the arguments fragmented and diluting their overall impact. To improve the manuscript's focus, I recommend reorganizing certain portions of the text.

Here are some examples:
a. In lines 747-758, the author's justification for the rarefaction approach would be better suited to the discussion section, as the methods should focus strictly on procedures.
b. Figure 5 panel a, the displayed equations would be more appropriately placed in the methods section.

5. The manuscript relies heavily on multipanel figures for its narrative. I recommend that the authors critically review each panel to streamline the visuals by removing non-essential elements and correcting any errors.

Here are some specific examples:

Fig 1c: A1 and A2 are the same color.
Fig 1f: The "Control" and "Treatment" labels appear to be reversed on the blue bar plot.
Fig 1g: The overall presentation is currently very confusing and requires clarification.
Fig 4a: The figure legend lacks sufficient explanation; the terms "Dam" and "Pup" are not defined.
Fig 5b: For clarity, I suggest removing the "DESeq2 TSS sensitivity" column from the table and instead labeling the corresponding curve directly within the plots.

(Remarks on code availability)

Version 1:

Reviewer comments:

Reviewer #1

(Remarks to the Author)
I appreciated the authors' careful responses to my comments from the review and corresponding updates to the manuscript. This applies especially to the clarification re: the two organisms used to build the mock MTX sample having been grown separately and transcriptionally frozen prior to being mixed. This is very sensible, but my major comment #1 was based on a misread of the procedure, so having it clarified in the main text will likely help other readers avoid similar confusion. I have no new concerns about the manuscript and look forward to seeing it in its final form.

(Remarks on code availability)
I looked through the contents of the repo and it looks thorough and useful to future researchers building on this paper. I did not install or run any of the code myself.

Reviewer #2

(Remarks to the Author)
I recommend the publication of the revised manuscript titled "Enhancing inference of differential gene expression in metatranscriptomes from human microbial communities" in Nature Communications.

(Remarks on code availability)

**Open Access** This Peer Review File is licensed under a Creative Commons Attribution 4.0 International License, which permits use, sharing, adaptation, distribution and reproduction in any medium or format, as long as you give appropriate credit to the original author(s) and the source, provide a link to the Creative Commons license, and indicate if changes were made.
In cases where reviewers are anonymous, credit should be given to 'Anonymous Referee' and the source.
The images or other third party material in this Peer Review File are included in the article's Creative Commons license, unless indicated otherwise in a credit line to the material. If material is not included in the article's Creative Commons license and your intended use is not permitted by statutory regulation or exceeds the permitted use, you will need to obtain permission directly from the copyright holder.
To view a copy of this license, visit https://creativecommons.org/licenses/by/4.0/

REVIEWER #1

MAJOR COMMENTS

Point 1. I will say from the outset that I thought the authors' approach of using monoculture DE to establish a gold standard for community DE was very clever, and I can't really imagine a better way of doing this. That said, unless I'm missing something, there's a huge assumption here that the monoculture bug doesn't change its transcriptional program in the community context (i.e. that DE'ed vs. non-DE'ed genes persist as a reasonable gold standard from monoculture to community context). Indeed, some of the findings of the gnotobiotic experiment underscore that bugs do different things when community structure changes. I think this can be largely addressed as part of an expanded "strengths and weaknesses" discussion vs. purely synthetic approaches (which are more controlled but less realistic). If there IS any way to estimate the stability of the DE lists, that would be interesting. Many other aspects of potential monoculture vs. community changes (e.g. effect of total transcript pool) WERE very carefully controlled in the study - well done.
in the study - well done.

*Our response* - We appreciate the reviewer's comment. We believe this assumption is reasonable based on the way the samples were prepared. We have added the following sentence to Results (lines 208-9 in Lee et al Manuscript [no tracked changes version]):

"*Because the two organisms were grown separately and transcriptionally 'frozen' prior to being mixed, the same set of differentially expressed P. copri genes should be identifiable in both the monoculture and mixture contexts.*"

Similarly, in analyses using the original, non-rarefied metatranscriptomes, although the DE genes themselves should persist between mixtures and monocultures, the ability to infer them in the face of total transcriptional output changes notably does not persist due to the relative nature of sequencing counts (**Fig. 1b,c**). We have noted this in the text (lines 286-289): "*Although this prevents consistent identification of 'ground truth' gene upregulation between monocultures and mixtures, we leveraged this feature of the metatranscriptomes to determine whether methods could recover upregulated genes in both conditions despite global transcription rate changes, as is possible in single-organism analysis.*"

**Point 2**. I was surprised by some of the differences in method performance between the synthetic and mock community data (especially variability in robustness to species abundance effects), and I wonder to what extent this was influenced by the difference in synthetic vs. mock sample sizes? The synthetic datasets had N=100 samples whereas it sounded like the mock communities had N=6 replicates, which I assume means N=6 vs. 6 in testing? (Notably these details were hard to find in the text, despite their importance.) I don't expect the authors to scale up to N=100 mock community replicates, but they could easily subsample the synthetic data down to N=12 (or whatever is appropriate) for a fairer comparison. Incorporating more AUC-based comparisons might also help, as this softens the influence of methods' dependence on N for achieving $p<0.05$.

***Our response*** - To address this as well as a related point from Reviewer 2, we have used the published simulation framework to generate additional simulated datasets with i) equivalent sample sizes of 6 per condition and ii) equivalent sample sizes and equivalent complexity of two species with the relative abundances in our mock communities. We quantify performance on these new simulated data in **Supplementary Fig. 7.** We write (lines 301-8):

Washington University School of Medicine, MSC8510-0041-04, 4515 McKinley Ave., St. Louis, MO 63110, (314) 362-7243, *Fax:* (314) 362-7047, jgordon@wustl.edu, website: http://gordonlab.wustl.edu

"*In both the complex (100 species) and two species synthetic communities, MTXmodel exhibited the highest sensitivity and best differential expression classification with the highest AUC (**Supplementary Fig. 7c,d**). The performance difference between MTXmodel and taxon-scaled DESeq2 was much more pronounced in the two-species communities, where MTXmodel demonstrated near-perfect sensitivity and AUC at high simulated relative abundances (**Supplementary Fig. 7d**). These results indicate that the relative performance of methods in synthetic datasets does not depend on sample size nor community complexity, and that the simulation framework likely encodes assumptions (e.g. direct scaling of RNA expression by DNA abundance) that inflate the performance of MTXmodel.*"

We also thank the Reviewer for observing that sample size details were absent in the main text. We have added the sample size for simulated data ("*Six published simulated datasets, each with 100 samples split into cases and controls*", lines 120-1) and mock communities ("*n=6 per growth condition per relative abundance*", line 196) to *Results*.

**Point 3**. I'm confused by the behavior of DESeq2 CSS DNA in the Fig. 1f ROC plots. It seems to be near optimal in 4/5 plots, but that's not reflected in the nearby heatmaps (where it's less of an outlier). There was a note in methods about DESeq2 AUCs being inflated that seemed like it might be related (in addition to being generally concerning from a benchmarking standpoint).

***Our response*** - We have updated our AUROC calculations to account for the fact that methods often skip differential expression testing in contexts with insufficient counts data (e.g. all or many 0s). Previously, AUROC was only calculated using the subset of genes which underwent differential expression testing, which inflated performance of methods which filtered out these challenging prediction cases. We have now instead imputed *P*-values of 1 (no predicted differential expression) for these missing DE tests, which has correspondingly lowered the AUROC of DESeq2 CSS DNA. We note this in the Results (lines 162-5): '

"*Notably, despite the high precision of their predictions, DESeq2 CSS DNA and TSS DNA exhibited lower AUCs than taxon-scaled DESeq2 without DNA covariates due to having a larger fraction of missing differential expression predictions (**Fig. 2g**; see Methods).*"

We have also added the following sentences to *Methods* (lines 552-7):

"*In cases where differential expression testing was skipped for genes or genomes with insufficient abundance, P-values of 1 (no differential expression) were imputed to prevent inflation of method performance. This is most apparent for DESeq2 CSS DNA, which exhibited high precision for its predictions but skipped testing for large fractions (45-70%) of the datasets (**Fig. 2c; Supplementary Table 1b**), and in the 'true-combo-bug-exp' dataset where MTXmodel strict zero-filtering resulted in skipped testing for 30% of genes.*"

MODERATE COMMENTS

**Point 4**. Related to the major comment above about monoculture vs. community DE, I appreciated that the authors didn't use a single method to establish monoculture DE, as this would have some of the same simulation method vs. analysis method concerns that the authors criticized in the context of purely synthetic evaluation. That said, it is worth discussing how focusing on a consensus monoculture DE genes set might affect community-level evaluations, and indeed if this consensus might miss certain classes of true biological DE.

***Our response*** - We originally did use a single method to establish monoculture DE (but showed that all individual methods produce consistent results in **Fig. 3c**). However, we appreciate this as a shared concern of Reviewer 1 and Reviewer 2 (see their comment on potential methodological bias for DESeq2-based

approaches). Therefore, we have now additionally used ‘consensus’ sets of differentially expressed genes, defined as the union of all ‘true positive’ and ‘true negative’ genes defined across the methods in **Fig. 3c** when applied to the monoculture samples. We calculate performance metrics using these consensus differentially expressed (and non-DE) genes in a new **Supplementary Fig. 5** and show that method robustness and susceptibility to confounders are identical to results using DESeq2 alone to define the true positive and true negative genes. These changes are described in the following paragraph that has been added to the revised manuscript (lines 276-83):

“*To ensure that using DESeq2 CSS to define true positive and true negative genes was not a source of methodological bias favoring performance of DESeq2-based methods, we defined separate ‘consensus’ benchmarking gene sets (571 true positives and 1,304 true negatives) taken as the union across the respective sets defined by each tested method when applied to the monoculture samples (**Supplementary Fig. 5a**). We then separately calculated performance metrics using these gene sets. Sensitivity, specificity, precision, and AUC were slightly lower across methods because no individual method completely captured these consensus sets (**Supplementary Fig. 5b-e**). However, the robustness and susceptibility of methods to low relative abundance, differential abundance, and varying prevalence were unchanged (**Supplementary Fig. 5e-g**).*

**Point 5**. When reporting benchmarks / making recommendations based on species abundance, I strongly recommend reporting an estimated coverage depth instead of (or alongside) relative abundance (RA). The former does not depend on sequencing depth, while the latter does, and sequencing depth was not always immediately referenced alongside RA values for context (and would require additional math from the reader to translate anyhow). As context, I strongly suspect that something like loss of sensitivity at low relative abundance has more to do with (e.g.) only 50% of the genome being covered, and if you increased depth by 10x (without changing relative abundance) you might see the problem alleviated. If that's NOT the case, and (say) RA effects have more to do with community complexity vs. genome coverage, THAT is worth emphasizing.
bundance has more to do with (e.g.) only 50% of the genome being covered, and if you increased depth by 10x (without changing relative abundance) you might see the problem alleviated. If that's NOT the case, and (say) RA effects have more to do with community complexity vs. genome coverage, THAT is worth emphasizing.

***Our response*** - We agree that (i) estimated depth, and not species relative abundance, is the key determinant for successful DE and (ii) deeper sequencing of a sample with the same relative abundance profile should increase inference of differential expression. Our current estimates for sensitivity are reported using number of reads mapped to an organism (lines 316-9):

“*Additionally, for the sample size of six replicates used here, we recommend minimum sequencing efforts of approximately $10^6$, $10^5$, $10^4$, and $10^3$ reads mapping to an organism to respectively provide sufficient coverage for inference of 80%, 50%, 20%, and 5% of its differentially expressed genes.“* To facilitate these comparisons, average *P. copri* mapped reads have also been added as a additional label for the mock community experiment design and performance metrics in **Fig. 3a**,**f**, and **g.**

We opted to report the number of mapped reads instead of genome coverage metrics. Although coverage (and theoretically, DE inference) will be lower for larger genomes with equivalent sequencing depth, the length of reads (part of coverage calculation) is not necessarily informative because the reads are used as tags/counts and not for genome assembly. As an example, having 10M 75bp reads vs 10M 150 bp reads doesn’t give you a 2x difference in DE inference if the reads are all being reliably mapped.

**Point 6.** The fact that the DEseq2-inspired methods only seem to be optimal at very high prevalence is limiting from a human epidemiology POV. I appreciated that the authors noted this at least once (i.e. stressing that these methods are best for in vitro / gnotobiotic work), but that wasn't super consistent with those methods coming across as the authors' main recommendation for MTX DE (assuming I was reading into that

correctly?). The human microbiome application at the end gets into this some with the sample:genome filtering suggestions, which were interesting and made sense, but by that point the authors were operating without a synthetic OR mock gold standard for unbiased evaluation.

***Our response*** **-** We appreciate that we had not previously shown that the sample filtering strategy applied in the human study analysis could overcome low prevalence with a gold standard unbiased evaluation. Therefore, we have added mock community benchmarking of depth and detection filtering using the same thresholds as the human analysis to **Supplementary Fig. 9**. Lines 408-11 of the revised manuscript now state

"*We applied these thresholds to our mock communities and found that while they decreased inference at low relative abundances, they increased robustness to confounding low prevalence (**Supplementary Fig. 9j-l**). We subsequently used these thresholds for comparisons of exclusion and inclusion of low information samples in the human study.*"

We now state in the Discussion (lines 465-9) note that zero-inflation remains a persistent challenge in metatranscriptomic analysis:

"*We recommend taxon-scaled DESeq2 for metatranscriptomic analyses where the prevalence of organisms of interest is high. We demonstrate that sample exclusion using genomic metrics of depth and detection can mitigate the confounding effects of low prevalence of taxa in human studies. However, zero-inflation of metatranscriptomic data from human studies remains a significant challenge; as such, new statistical methods designed explicitly for metatranscriptomic differential expression analyses and tested using the framework described here are needed.*"

**Point 7**. If a difference in Ns does not explain the synthetic vs. mock benchmarking differences (see major comment above), it would be nice to at least speculate on what MIGHT explain the differences for the purposes of improving the construction of future synthetic datasets and/or MTX DE methods.

*Our response* - As noted above, MTXmodel outperforms taxon-scaled DESeq2 in our new simulated datasets with 6 samples per group and either equivalent complexity or two-species with abundances mirroring the mock communities.  While we cannot determine definitively which specific aspect of the simulation framework favors the performance of MTXmodel, we believe it is likely due to direct linear scaling of underlying RNA expression levels by corresponding DNA abundance levels. This is likely an unrealistically clean assumption for quantification of metagenomes and metatranscriptomes in real datasets.

**Point 8**. I applaud the authors for the amount of benchmarking detail in Fig. 2 and 3 (and their SI analogs). However, it might be nice to also find a couple of views that would facilitate easier "at-a-glance" comparison between the various methods. For example, maybe a couple of representative AUC bar plots in Fig. 3 that illustrate the authors' core messages about robustness to differential abundance vs. prevalence.

***Our response*** **-** We appreciate the Reviewer's comment about the density of these two figures. For simulated data analysis in **Fig 2**, sensitivity, specificity, and precision heatmaps for organisms grouped by relative abundance are now presented in **Supplementary Fig. 2h-j**. For mock community analyses in **Fig. 3**, we have substituted the sensitivity, specificity, and precision metrics with summary ROC AUC heatmaps for each set of comparisons. The original TPR/FPR/PPV heatmaps are now shown in **Supplementary Fig. 4**.  We have specifically retained sensitivity for the low abundance comparisons in the main text figure (**Fig. 3f**) since this is the basis for our recommendations of sequencing depth per organism.

MINOR COMMENTS

**Point 9**. It would be nice to include a small description of the meaning of the different synthetic datasets to help with interpretation (in the spirit of the tables in Fig. 1a).

***Our response* -** We have added panel a to **Fig.** 2 describing characteristics of the simulated datasets.

**REVIEWER #2**

**Point 1 -** At the core of this manuscript is a benchmarking study using in vitro mock communities. The authors conclude that taxon-scaled DESeq2 performs best among the tested methods. However, the gold standard for comparison was also established using DESeq2 on a single-species "community" of P. copri—which, by its nature, is inherently "taxon-scaled." This raises a concern about potential methodological bias, as the gene set used for evaluation was selected by the very method that is regarded as the top performer. I recommend the authors dedicate a section of the discussion to explicitly address this concern in their manuscript.

I recommend the authors dedicate a section of the discussion to explicitly address this concern in their manuscript.

***Our response***: We have repeated benchmarking using consensus sets of differentially expressed genes defined across the tested methods to reduce potential methodological bias. We show that individual method performance under confounders (low relative abundance, differential abundance, and varying prevalence) are the same in a new **Supplementary Fig. 5**. See a related point and our response under Reviewer 1's Moderate Comments.

**Point 2.** While the mock community experiments involve mixtures of P. copri and E. coli at different ratios, the simulation studies do not appear to include a directly analogous dataset. Specifically, a simulated community replicating the simple two-species composition and known ratios used in the mock experiments would provide the most straightforward comparison. I recommend that the authors address this point in the manuscript, explaining their rationale for not including this most directly comparable simulation scenario in their study.

scenario in their study.

*Our response* **-** In brief, we generated simulated analogs to the mock communities with either 6 samples per group and the same 100 species complexity based on Human Microbiome Project datasets, or with 6 samples and two species whose abundance mirror those seen in the mock communities. We quantify performance on these datasets in a new **Supplementary Fig. 7**. See our response to Reviewer 1's Point 2.

**Point 3**. The authors employ rarefaction to address global RNA expression changes. This method should be introduced immediately following the discussion of genomic abundance (lines 78–87), as the two issues are presented as equally important in Figure 1b.

*Our response* - Rarefaction (and its limitations in the context of controlling genomic abundance for metatranscriptomics) is now described in the Introduction (lines 75-8): "*Rarefaction (sub-sampling of counts without replacement), historically used for bacterial 16S rRNA amplicon analyses, offers a potential solution to control for changes in genomic representation between conditions. However, the dynamic range of biological variance in organism abundances typically exceeds that of the technical variance in sequencing depths. Rarefaction also inherently compromises statistical power by reducing available information.*"

**Point 4.** The author's presentation blurs the boundaries between sections, making the arguments fragmented and diluting their overall impact. To improve the manuscript's focus, I recommend reorganizing certain portions of the text. Here are some examples: a. In lines 747-758, the author's justification for the rarefaction

approach would be better suited to the discussion section, as the methods should focus strictly on procedures.
b. Figure 5 panel a, the displayed equations would be more appropriately placed in the methods section.

***Our response***: We appreciate the Reviewer's comments. To improve the manuscript's focus, we have converted the following three sections to *Supplementary Notes* and substituted in their place summary paragraphs containing problem definition, methodology, and mainline results:

**Validation of mock community properties and methodology** - now Supplementary Note 1, lines 891-935)**.**
**Benchmarking with confounding transcription rate changes** - now Supplementary Note 2, lines 937-70)**.**
**Validation of cross-feeding interactions *in vitro*** - now Supplementary Note 3, lines 972-1008).

These three sections total 1,832 words. Accounting for the replacement summaries, their relocation to *Supplementary Information* has shortened the manuscript by approximately 1,300 words. These changes should emphasize, and make more accessible, the key results about method performance.

Note that our justification for the rarefaction approach has been moved to Supplementary Note 1 describing validation of mock community properties and the rarefaction and quantification approaches. The displayed equations in Figure 5a have been removed and put in Methods.

**Point** 5. The manuscript relies heavily on multipanel figures for its narrative. I recommend that the authors critically review each panel to streamline the visuals by removing non-essential elements and correcting any errors.

***Our response***: We appreciate this comment. We have made the following changes to figures to streamline the presentation:

**Fig.2h-j** showing performance metrics as a function of organism abundance in simulated datasets are now shown as part of **Supplementary Fig. 2h-j** and removed from **Fig. 2**.

**Fig. 3f-h** which showed TPR, FPR, and PPV for each mock community comparison with no differential abundance (f), differential abundance (g), or varying prevalence (h) have been replaced with ROC AUC heatmaps. The original TPR, FPR, and PPV heatmaps have been moved to **Supplementary Fig. 4**.

**Fig. 5** equations (panel a) have been moved to *Methods*.

Fig 1c: A1 and A2 are the same color.
This has been fixed

Fig 1f: The "Control" and "Treatment" labels appear to be reversed on the blue bar plot.
This has been fixed

Fig 1g: The overall presentation is currently very confusing and requires clarification.
We have changed underlying expression distribution from histogram to a probability density function and clarified the limit of detection as counts > 0.

Fig 4a: The figure legend lacks sufficient explanation; the terms "Dam" and "Pup" are not defined.
We have changed wording to mothers and offspring to reduce ambiguity.

Fig 5b: For clarity, I suggest removing the "DESeq2 TSS sensitivity" column from the table and instead labeling the corresponding curve directly within the plots.
We have now made changes to **Fig. 5b** (now 5a)
